# Comparing Eye-Tracking Metrics with the Driver Activity Load Index

**DOI:** 10.3390/jemr19020028

**Published:** 2026-03-05

**Authors:** Julia Bend, Markus Gödker, Elise Sophie Banach, Thomas Franke

**Affiliations:** 1 Faculty of Human and Social Sciences, Åbo Akademi University, 20500 Turku, Finland; 2 Faculty of Psychology, Ludwig-Maximilians-Universität München, 80539 Munich, Germany; 3 Institute of Multimedia and Interactive Systems, University of Lübeck, 23562 Lübeck, Germany

**Keywords:** perceptual load, cognitive load, driving, DALI, eye tracking, subjective workload assessment, human factors, driving simulator, situational complexity

## Abstract

This study investigated how perceptual workload in driving situations is captured by subjective ratings versus eye-tracking metrics. Fifty participants completed low- and high-complexity conditions while fixation behavior, blinks, and pupil diameter were recorded, and workload was assessed using the DALI scale. High-load scenes elicited longer fixations, fewer fixations per minute, reduced blinking, and increased pupil dilation, indicating elevated attentional demand. DALI scores increased with scene complexity and were negatively associated with fixation duration, demonstrating that participants’ subjective ratings were driven primarily by perceptual strain rather than cognitive effort. Eye-tracking patterns supported this interpretation: fixation-based indicators tent to reflect the cognitive component of demand, whereas DALI selectively tracked perceptual overload. Together, these results show that DALI is highly sensitive to visual density, and that eye-movement measures provide converging evidence for its specificity as a perceptual load instrument.

## 1. Introduction

Suboptimal cognitive functioning—such as lapses in attention or episodes of drowsiness—is widely recognized as a major contributor to traffic accidents and reduced driving performance. Recent findings from the Traffic Safety Culture Index indicate that 87.5% of drivers now view distracted driving as a more serious concern than in previous years, and 87.9% consider drowsiness to be a significant safety threat [1]. These public concerns align with ongoing research efforts aimed at improving road safety by monitoring drivers’ cognitive states, including mental workload, inattention, and fatigue [1,2].

A range of methods can be used to evaluate cognitive states, commonly grouped into subjective, behavioral, and physiological measures [3]. Subjective tools—such as the well-established NASA Task Load Index (NASA-TLX)—are frequently employed to capture perceived workload [1,4,5,6]. However, subjective ratings have important limitations: real-time data collection may interrupt task performance, and individuals are not always accurate judges of their own cognitive state [7]. People may misestimate levels of attention, workload, or drowsiness, which can lead to unreliable assessments. These challenges highlight the need for objective measures to complement self-reports and to support more accurate monitoring of drivers’ cognitive functioning for traffic safety.

A growing body of research has demonstrated that physiological signals offer a robust window into drivers’ cognitive and mental states. Methods such as electroencephalography and event-related potentials, cerebral blood-flow measures, electromyography, thermal imaging, heart-rate variability, blood pressure, and pupillometry have all been shown to reliably track changes in cognitive workload during driving [1,2,8]. These indicators typically respond to increases in mental demand even when traditional behavioral driving metrics—such as steering reversals and vehicle-control variability—remain unchanged [9,10].

A key advantage of physiological metrics is that they are largely involuntary and therefore less susceptible to conscious regulation compared with overt behaviors like speech, facial expressions, or self-evaluation. Furthermore, given that mental workload is a multidimensional and dynamic construct, relying solely on subjective reports such as NASA-TLX provides only a partial picture of the driver’s cognitive state. Prior work has emphasized that a combination of self-reporting, performance measures, and physiological indicators provides a more complete and accurate assessment of mental workload [1]. The integration of physiological data thus enhances the sensitivity of workload monitoring systems and improves the detection of moment-to-moment cognitive fluctuations that influence driving performance and safety [11].

Vision plays a central role in driving, as drivers rely heavily on continuous visual monitoring of the road environment and other traffic participants. Because of this, eye-movement behaviour has become an important source of information for assessing mental workload, fatigue, and visual attention during driving [2,12,13,14]. Moreover, improvements in camera technologies and computational processing have further advanced remote eye-tracking systems, making them practical, non-intrusive tools for real-time assessment of driver state, including fatigue and workload [13]. Numerous studies have demonstrated that eye-tracking metrics sensitively reflect task demands in both manual and semi-autonomous driving. For example, Yang and Kuo [15], Matton et al. [16], and Chen et al. [17] showed that gaze behaviour changes systematically under multitasking, and Yang et al. [15] reported that high distraction can produce extremely long off-road glances lasting more than 30 s.

In the present study, we examine how fixation-related metrics, blink behaviour, and pupil size vary in relation to drivers’ self-reported mental workload while navigating low- and high-complexity road sections in a driving simulator equipped with wearable eye tracking. Specifically, we analyse fixation duration, fixation rate (see Section 1.2), blink duration, blink rate (see Section 1.3), and pupil diameter (see Section 1.1) alongside the Driving Activity Load Index (DALI, [18]; see Section 1.4 and Section 1.5). We used DALI—rather than NASA-TLX—because it is purpose-built for driving, targets modality-specific (visual/auditory) demands, and is efficient for repeated within-session ratings, improving sensitivity to workload changes linked to eye movements. Our aim was to assess the correspondence between subjective workload ratings and objective eye-tracking indicators and determine which oculomotor metrics best reflect participants’ perceived mental workload.

### 1.1. Pupil

Pupil size is one of the most extensively researched physiological indicators of mental workload in eye-tracking studies [19,20,21]. Beatty [22] demonstrated that the amplitude of task-evoked pupillary responses increases systematically with processing load. When multiple oculomotor metrics are collected, pupil diameter often emerges as the most stable and reliable correlate of cognitive effort [6,23].

However, pupil dilation is influenced by more than task demands alone. In addition to cognitive workload, changes in pupil size can reflect emotional arousal and other higher-order processes, making it challenging to isolate the component of internal processing that drives the response [6]. Despite this lack of specificity, research suggests that pupil diameter is sensitive not only to high workload but also to underload: reduced arousal and boredom can decrease pupil size, whereas excessive demands may produce marked dilation associated with cognitive overload and frustration.

Task-evoked pupillary responses typically appear 100–200 ms after the onset of mental processing and diminish rapidly once processing ends [22]. By contrast, pupillary responses related to emotional or motivational factors tend to persist longer and influence baseline pupil diameter. A range of non-cognitive variables—ambient lighting, screen luminance [24], age, refractive errors, sleep deprivation, mental-health conditions, and substances such as caffeine or alcohol—can also modulate pupil size [6,25]. Because illumination has such a strong effect on the pupil light reflex, interpreting workload-induced pupil changes becomes more difficult when luminance is not carefully controlled or reported. Many studies therefore emphasize maintaining constant lighting conditions or highlight the potential confounding effects of illumination variability.

A key limitation of using pupil diameter as the sole marker of workload is its limited diagnostic value: it reflects the intensity of mental effort but does not indicate where in the processing stream the load originates, nor whether the underlying state is positive or negative [6,21]. For example, Mallick et al. [26] increased perceptual demands in a Tetris task by accelerating falling blocks, whereas Di Stasi et al. [27] induced cognitive load by requiring more effortful problem-solving strategies. Although these manipulations targeted different processing stages (perceptual vs. cognitive), both resulted in increased pupil dilation. As Liu et al. [21] argue, fixation-related parameters can complement pupil-based metrics by offering more direct information about the perceptual and cognitive processes driving workload—an approach that has also been highlighted in recent work [28].

### 1.2. Fixation

Fixations occur when the eyes stabilize on a specific location, allowing visual information to be processed at the fovea—the region of highest acuity due to its dense concentration of photoreceptors and specialised retinal circuitry [6,29]. Eye-tracking studies commonly quantify fixation behaviour using measures such as fixation duration and fixation rate, both of which are sensitive to changes in task demands.

Both cognitive load and perceptual load influence fixation patterns, but they do so in different ways. Perceptual load refers to the amount of bottom–up, stimulus-driven processing required by the visual scene, whereas cognitive load reflects top–down processing and higher-order operations [30]. Perceptual load can be manipulated by increasing the number or complexity of visual elements, while cognitive load is typically elevated through tasks that require memory, strategy, or mental imagery [31]. When perceptual demands exceed the available perceptual capacity, viewers tend to make shorter and more frequent fixations as they attempt to scan and integrate a larger amount of sensory information. In contrast, high cognitive load is associated with longer and fewer fixations, reflecting deeper processing during each fixation and reduced capacity to filter irrelevant information [21].

To disentangle these mechanisms, Liu et al. [21] systematically manipulated cognitive and perceptual load in a puzzle-solving video game. Their results confirmed that higher cognitive load produced longer fixation durations and reduced fixation frequency, whereas higher perceptual load caused shorter durations and increased frequency [21]. Similar patterns have been reported in other gamified and simulation-based tasks: for example, Marandi’s visual memory task showed longer fixations and fewer gaze shifts as memory demands increased, and Schirm et al. [32] observed reduced fixation frequency and a trend toward longer duration during effortful VR language comprehension. These outcomes are consistent with increases in cognitive demand. Conversely, tasks characterized by rapid stimulus changes—such as Mallick et al.’s [26] manipulation of falling-block speed in Tetris—typically elicit shorter and more frequent fixations, reflecting increased perceptual load. Di Stasi et al. [27] similarly demonstrated that stimulus-driven firefighting strategies produced perceptual load patterns, whereas strategic, prediction-based approaches elicited cognitive load patterns. Recent driving studies also support the value of fixation metrics, with Weifeng et al. [33] and Sun et al. [34] showing that fixation duration and pupil size reliably signal driver fatigue.

Fixation detection itself poses methodological challenges, especially in head-mounted eye tracking where head and body movements introduce noise [35]. While many studies rely on velocity-threshold algorithms to classify fixations, this approach can be less stable in mobile setups. Therefore, in the current study, we adopted a functional definition of fixation: any eye movement that stabilizes a visual target on the retina was treated as part of a fixation [36]. Under this definition, microsaccades remain embedded within fixations and do not fragment them into shorter events [35]. The fixation rate was calculated as the number of fixations divided by trial duration (in seconds), a standard measure in eye-tracking research.

### 1.3. Blinks

Blinking is an automatic reflex that briefly closes both eyes to maintain ocular lubrication, protect the cornea, and clear the visual surface. A typical adult blinks roughly 12 times per minute, with each blink lasting about one third of a second [37,38]. Common blink-related metrics include blink rate, blink duration and blink latency [13,39,40,41]. The blink rate—which counts the frequency of blinks per minute or second—is often used because it only requires detecting blink occurrences rather than capturing blink duration or amplitude. However, substantial variability has been documented both across and within individuals [42]. For example, Zaman and Doughty [43] showed that blink rate can fluctuate considerably even within a few minutes, highlighting the importance of timing when collecting blink data.

Many studies have linked blink behaviour to changes in mental workload and fatigue. Tsai et al. [44] observed that blink rate increased when drivers performed an additional auditory task, and Van Orden et al. [45] reported blink-rate increases associated with tracking errors. In an occupational setting, Li et al. [46] found that fatigue increased blink rate while reducing pupil size and narrowing the range of visual attention. Even so, the direction of blink-rate changes is not uniform across studies, largely because blink behaviour depends on the type of task demand. During highly visual tasks, blink rate is often suppressed to maintain continuous visual input, whereas tasks requiring internally directed attention tend to increase blinking [6].

Blink duration also reflects complex interactions between perceptual and cognitive processes. A recent review by Skaramagkas et al. [23] found that most studies reported decreases in blink frequency with higher cognitive workload and increased visual attention demands. The inconsistencies reported in the literature appear to stem from differences in task characteristics. In piloting studies, for example, Veltman and Gaillard [47] found that blinks decreased when pilots needed to process more visual information, regardless of air traffic control difficulty. Conversely, when cognitive demands exceed available resources, reduced concentration or fatigue may lead to increased blinking [42].

Blink metrics are also associated with attentional performance and physiological arousal. Both blink rate and blink duration correlate with subjective sleepiness scores and EEG indicators of reduced alertness [48]. In tasks that require sustained visual engagement, such as reading or monitoring displays, blink suppression is common. In contrast, prolonged blink duration is a strong indicator of fatigue, making it more informative for detecting drowsiness than blink rate alone [48].

### 1.4. NASA-TLX and DALI

In addition to the increasing use of autonomic and gaze-based indicators, subjective ratings and performance metrics remain commonly used tools for assessing mental effort. In their meta-analysis of gaze and pupil measures in gamified and simulated sensorimotor tasks, Gorin et al. [6] noted that many studies combine all three types of assessments. Performance indicators typically include measures such as accuracy, completion time, number of attempts, and task scores, whereas the NASA Task Load Index (NASA-TLX) is the most widely used subjective instrument [4]. Originally developed for evaluating operators of human–machine systems, NASA-TLX has since been adopted across a broad range of domains. The instrument comprises six subscales—mental, physical, and temporal demand, effort, performance, and frustration—and traditionally yields a weighted composite score [5,49]. Many researchers now analyse subscales individually to determine which aspects of workload are most sensitive to task manipulations [6]. For instance, Marandi et al. [50] found significant differences between difficulty levels and reported that mental and temporal demands were the most responsive in their computer-based task.

Most studies administer the NASA-TLX immediately after each condition or task segment [50]. This raises the issue that any attempt to collect subjective ratings during task execution would interrupt performance—an especially critical concern in contexts involving a flow state. In tasks capable of inducing a flow state, characterised by deep immersion and continuous engagement, prompting participants for subjective judgments during the activity would break this immersion and disrupt the task. However, administering NASA-TLX only after task completion may introduce limitations related to retrospective recall. To reduce intrusiveness and response burden, some researchers use shorter instruments either in addition to or instead of the NASA-TLX [6].

The Driving Activity Load Index (DALI) is a domain-specific adaptation of the NASA-TLX, designed to capture the multidimensional aspects of workload in driving [18]. The DALI uses the same rating and weighting procedure as the NASA-TLX but replaces several dimensions to better reflect the perceptual and cognitive requirements. Pauzié [18] argues that certain elements of the NASA-TLX, such as physical load or the general formulation of mental load, are not sufficiently aligned with the characteristics of modern driving, where physical effort should be minimal for an experienced driver and mental load should be distributed across the modalities of perceptual and cognitive processes. The DALI introduces six driving-specific workload dimensions based on expert interviews, item generation and validation studies: effort of attention, visual demand, auditory demand, temporal demand, interference and situational stress [51].

Because the underlying structure and scoring principles are consistent with the NASA-TLX, the DALI is theoretically compatible with the original instrument while providing enhanced sensitivity to the context of driving. Empirical studies comparing both measurement methods demonstrated that they generally correlate, suggesting that they assess shared aspects of workload, but the DALI appears to be most suitable for the driving context [52,53]. For this reason, the DALI can be used as an alternative measure in studies dealing with workload while driving.

### 1.5. Correlation Between NASA-TLX and Eye-Tracking Measurements

The interplay between subjective, behavioural, and physiological indicators remains complex and not fully resolved. Relying solely on subjective ratings and performance metrics risks missing important aspects of effort and workload: subjective judgments are susceptible to bias and cannot be collected unobtrusively in real time, while behavioural measures do not directly quantify cognitive effort. Physiological indicators—particularly those derived from eye movements—offer a complementary and more objective perspective on mental workload, enriching and extending traditional assessment approaches.

A growing body of research has examined how subjective workload ratings, particularly NASA-TLX scores, correspond with physiological and oculomotor indicators of mental effort. Several studies report meaningful associations between NASA-TLX and pupil-based measures. For example, Devos et al. [54] showed that task-evoked pupillary responses were significantly correlated with NASA-TLX ratings when assessing cognitive workload in older adults. Other work highlights more complex, non-linear relationships. In Strauch et al.’s [55] Pong game experiment, pupil dilation followed an inverted U-shaped pattern across difficulty levels: moderate difficulty elicited the largest dilation. Subjective ratings of enjoyment, focus, and engagement increased with this pupillary response, whereas boredom showed the opposite trend. A similar dissociation was reported by Wanyan et al. [56], where pupil dilation again exhibited an inverted U-shaped pattern, but NASA-TLX scores increased linearly with difficulty, and performance decreased linearly. This demonstrates that subjective workload does not always track the same non-linear patterns observed in physiological measures. The distinction between perceptual and cognitive load further complicates the relationship between subjective and oculomotor metrics. In a controlled comparison, Liu et al. [21] showed that increases in cognitive load—but not perceptual load—led to higher NASA-TLX scores and reduced performance. However, fixation-based metrics were sensitive to both types of load but in different ways: cognitive load increased fixation duration and reduced fixation frequency, whereas perceptual load produced the opposite pattern. These findings underscore the value of eye-tracking data as a complement to self-reporting, and they highlight that the type of demand imposed by the task critically shapes how workload is captured.

Consistent with this view, Chen et al. [17] investigated workload differences across three levels of vehicle automation (manual driving, partial automation, high automation) using both NASA-TLX and eye-tracking measures. Participants completed three types of secondary tasks—visual–verbal, auditory–spatial, and auditory–verbal—under each automation level, yielding nine conditions in total. Manual driving produced the highest workload, partial automation imposed moderate demand, and high automation generated the lowest workload. NASA-TLX scores captured these differences, while eye-movement measures provided additional insights into how perceptual and cognitive demands varied across automation levels. Among the physiological indicators, pupil diameter change and 3D gaze entropy emerged as the most reliable markers of MWL in their study. Pupil size change showed a positive correlation with NASA-TLX scores, confirming its sensitivity to subjective workload, whereas gaze entropy increased with task difficulty, suggesting greater attentional variability and more complex visual-search behaviour under higher demands. Fixation behaviour also varied systematically with automation level. Overall, fixations were longer and less frequent in high automation compared with manual driving, indicating that increased automation altered visual sampling strategies. In the visual–verbal task, fixation duration decreased from 0.06 s (manual driving) to—0.03 s (high automation), while the fixation number remained stable. In contrast, in the auditory tasks, the fixation duration increased while the fixation rate decreased under higher load, indicating deeper cognitive processing and stronger working-memory engagement. Chen et al. [17] further reported that fixation duration in the auditory secondary tasks was significantly positively correlated with MWL, supporting the interpretation that longer fixations reflect increased cognitive effort rather than perceptual strain.

Zheng et al. [49] examined associations between NASA-TLX scores and blink metrics among surgeons during laparoscopic operations. Although no significant correlations were found when analysing all participants together, dividing surgeons into low- and high-blink-rate groups revealed clear differences: individuals who blinked less frequently reported higher frustration and higher overall workload. The authors noted that this subgrouping limits generalisability, but the pattern nevertheless illustrates that blink behaviour can capture workload variations that subjective NASA-TLX ratings alone may not fully reflect. In line with previous research, we hypothesise that DALI scores will be positively associated with established eye-tracking measures of mental workload.

## 2. Materials and Methods

### 2.1. Participants

A total of 105 people took part in the driving simulator experiment. Recruitment was carried out via the University of Lübeck’s email distribution list, forums in the university’s learning management system and social media. Eligible participants were at least 18 years old with a valid driving licence and sufficient knowledge of German. Participation was voluntary and required written consent. Participants received either €18.62 (based on the German minimum wage) or, in the case of psychology and media informatics students, optional academic credits as compensation. The study was approved by the Ethics Committee of the University of Lübeck (tracking numbers 2023-680 and 2023-680_1). 

Four people ended the experiment prematurely due to simulation sickness, and a further eight datasets had to be excluded due to technical problems or irregular procedures (e.g., major display failures). Five additional datasets could not be taken into account due to technical malfunctions during driving data logging. With regard to the eye-tracking data, a homogeneous database could only be ensured after the introduction of a new eye tracking system (Pupil Labs Neon), as the previous setup had major technical failures. For this reason, we obtained eye-tracking data from 53 individuals from the 88 datasets, predominantly obtained using the new eye-tracking recording setup. Three additional datasets were unusable due to incompatible glasses worn by the participants, resulting in a final eye-tracking sample size of *N* = 50.

Participants were between 18 and 38 years old (*M* = 23.0, *SD* = 4.0). Thirty-six individuals (72%) identified as female, 14 (28%) as male; no one identified as diverse or did not specify (0%). The average driving experience with cars was *M* = 45,876 km (*SD* = 144,593, *N* = 48; due to a survey error, not all data was available). In total, 22% of participants already had at least 50 km of experience with battery electric vehicles, with an average of *M* = 801 km (SD = 1056).

### 2.2. Apparatus

**Figure 1:**
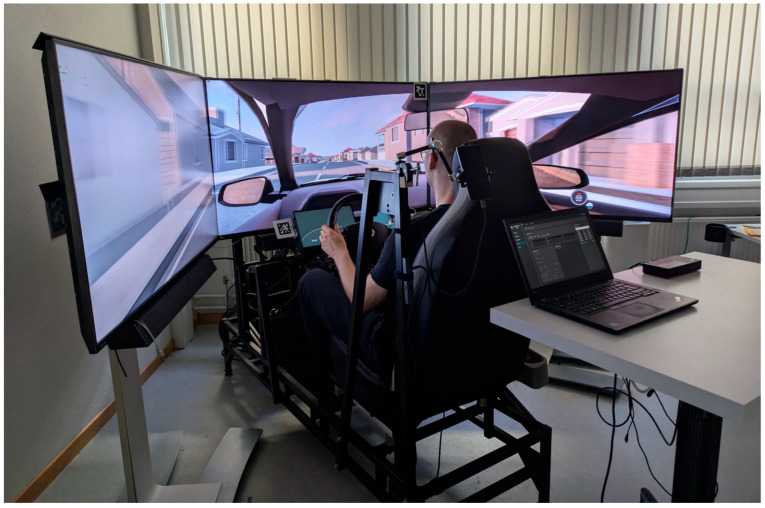
Setup of the EcoSimLab EV driving simulator.

The experiment was conducted in the EcoSimLab electric vehicle (EV) driving simulator at the Institute of Multimedia and Interactive Systems, University of Lübeck (see Figure 1). The simulator modelled a Renault Zoe EV as the reference vehicle within the EcoDrivingTestPark environment [57]. This virtual test track consists of seven distinct driving scenarios, each designed to elicit energy-relevant driving maneuvers. The scenario duration averaged 77.5 s (*SD* = 10.0, range = 55.8–123.6 s), and the scenario complexity was deliberately varied to capture a wide range of operational ecodriving situations. The simulator hardware comprised three 55-inch and 120 Hz displays, arranged to create a 180° field of view. Participants sat in a car seat mounted on a Fanatec rig, including pedals and a wheelbase, combined with a compact steering wheel. Both steering wheel and pedals were equipped with force feedback to enhance realism. Simulation was implemented using BeamNG.tech (Version 0.25.0.0), a research-focused variant of the BeamNG.drive engine [58].

The EcoDrivingTestPark was organized into map sectors embedded within a BeamNG.tech environment. Each sector began and ended with a curved tunnel containing a teleportation portal, which allowed seamless transitions between sectors without perceptible visual discontinuities. This setup enabled flexible randomization of sector order while maintaining an uninterrupted driving experience. To systematically address task demands, sectors were classified into high- and low-complexity blocks. Situation complexity was determined using the SAFE framework, which evaluates behavioral requirements for information processing and vehicle handling [59,60]. On this basis, Banach & Gödker [61] developed a short rating questionnaire derived from the SAFE’s core dimensions. They had independent experts in traffic and engineering psychology assess the sectors of the EcoDrivingTestPark in terms of their complexity using sector videos and the questionnaire. The key determinants of situational complexity used were (1) time pressure, (2) required accuracy, (3) number of requirements, (4) the share of conscious actions required, and the behavioral demands for (5) information processing and (6) vehicle operation, as well as an (7) overall complexity rating. The expert ratings showed high internal consistency and interrater agreement, confirming that the SAFE-based items accurately distinguished between the sectors. Clustering of ratings then allowed classification of the scenarios into two complexity blocks (high and low). Sectors 4, 5, 6, and 7 were the high-complexity sectors; sectors 1, 2, and 3 were the low-complexity sectors. High-complexity sectors were characterised by sharper turns, greater number of task demands and higher accuracy requirements while low-complexity sectors showed fewer external demands, minimal required decision-making and lower time pressure.

### 2.3. Materials and Measures

Subjective workload during driving was assessed using the Driver Activity Load Index (DALI; [18]). We used a German adaptation of the DALI and slightly adjusted item wording to match the present simulation context [51]. Participants rated six dimensions, effort of attention, visual demand, auditory demand, temporal demand, interference, and situational stress, on a 6-point Likert scale ranging from 0 = very low to 5 = very high. Cronbach’s α was 0.86 in both complexity conditions, indicating good internal consistency.

Eye-tracking measures were recorded using Pupil Labs’ Pupil Neon mobile eye-tracking glasses (Pupil Labs GmbH, Germany, Berlin). The system included an egocentric scene camera (1080 × 1088 px, 30 Hz) and two infrared (IR) eye cameras capturing each eye at 200 Hz. Participants were allowed to move their heads naturally. We analyzed the blink rate per minute, blink duration in milliseconds, fixation rate per minute and fixation duration in milliseconds (see Section 1.1, Section 1.2 and Section 1.3 for more details). As an additional exploratory indicator, average pupil diameter (mm) was analyzed descriptively but excluded from the main confirmatory models due to the absence of a pre-task baseline and potential luminance confounders.

### 2.4. Data Processing

To ensure reliable identification of blinks, only timepoints where both eyes were simultaneously undetected were considered. The dataset was restricted to samples recorded while participants were actively engaged in the experimental tasks, with all instructional and transition screens excluded. Further filtering retained only intervals during which both gaze signals were missing.

For blink detection, both lower and upper duration thresholds were applied to sequences of missing pupil data. The lower threshold was set to 100 ms, following the Pupil Labs blink detection algorithm and consistent with physiological boundaries reported in previous research [2,38,62,63,64].

Many researchers adopt blink duration thresholds between 80 and 160 ms, as this interval aligns well with typical spontaneous blink rates, although the optimal setting may vary depending on study design and recording equipment. For example, Hollander and Huette [38] used a 160 ms threshold, Frank et al. [62] and Huette et al. [63] each applied 100 ms, and Van Orden et al. [64] used 83.3 ms. The 100 ms threshold adopted here is widely used in eye-tracking research because it effectively distinguishes genuine blinks from short signal interruptions or occlusions, capturing natural blink behavior while minimizing false detections caused by noise or brief signal losses. Data losses exceeding 1000 ms were excluded, as such prolonged gaps likely reflected microsleeps or other non-task-related interruptions, thereby preserving the validity of blink-related measures.

For fixation detection, we employed the velocity-based I-VT (Identification by Velocity Threshold) algorithm, which classifies each gaze sample as a fixation or a saccade depending on whether instantaneous velocity is below or above a defined threshold [65,66]. Although effective under static viewing conditions, I-VT can be sensitive to head motion in head-mounted eye tracking. To mitigate this, we used the adaptive velocity-threshold algorithm implemented in Pupil Labs, with a minimum velocity threshold of 1200 px/s, a gain factor of 0.8, and a minimum fixation duration of 70 ms [35].

As a post-processing step, adjacent fixations separated by less than 2° of visual angle and 50 ms were merged to treat short microsaccadic interruptions as continuous fixations, consistent with established recommendations for mobile eye-tracking analysis [35]. No upper limit on fixation duration was imposed.

### 2.5. Procedure

The study used a repeated measures design in a driving simulator. Each participant completed both complexity blocks. At the beginning, participants were welcomed, informed about the procedure, and asked to provide written consent before completing an initial questionnaire. Subsequently, an instructional video introduced them to the overall study procedure. To gain familiarity with the simulator, they completed a four-minute tutorial drive that included various speed limits, curves, gradients, and descents. A second instructional video then explained the objectives and rules of the EcoDrivingTestPark. Participants were instructed to drive as energy-efficiently as possible, obey all German traffic regulations, follow the signposted route to the fictional city of Simnitz to avoid navigational errors, reach each sector’s destination within the predefined time limit (calculated as the time needed when driving at 90% of the sector’s speed limits), and respond to short online prompts whenever passing through teleportation tunnels.

Before entering the experimental drive, participants performed a practice drive on two non-experimental sectors of the EcoDrivingTestPark (not part of the present analyses) to become accustomed to the tasks. They then completed both complexity blocks, with a randomized order of complexity blocks and sectors. In each tunnel, the test subjects answered two live questions. Between the blocks, the test subjects answered the intermediate questionnaire. At the end of all drives, participants completed a final questionnaire. The full experimental procedure lasted approximately 90 min per participant. 

### 2.6. Overview of Questionnaires

In addition to the DALI, which is the primary subjective measure in this study, several other questionnaires were completed during the experiment. The initial questionnaire covered prior experience with ecodriving displays, driving simulators and gaming, ecodriving motivation and knowledge, and driving experience with internal combustion, hybrid, and battery electric vehicles. The short online prompts assessed the participants’ perceived understanding of current and future vehicle energy consumption, as well as cognitive demands regarding information intake, processing and action regulation. The intermediate questionnaire included the DALI and additional measures assessing perceived situational complexity and energy dynamics awareness. Between the baseline and the experimental phase, further questionnaires on the topics of action regulation, mental model and self-efficacy were completed. The final questionnaire collected demographics and personal characteristics, as well as the action regulation, mental model and self-efficacy questionnaires and user experience and system usability scales. For more detailed descriptions of selected questionnaires and their conceptual rationale, see [67], which reports analyses of energy-related situation awareness and ecodriving performance based on data from the same experiment.

### 2.7. Analyses

Only the experimental drives from the baseline phase were used in the statistical analysis (a total of seven sectors). These were divided into a high-complexity block (sectors 4, 5, 6, 7) and a low-complexity block (sectors 1, 2, 3) based on their original clustering (see Section 2.2). For each measure (eye-tracking measures and DALI) separate linear mixed-effects models were fitted with condition (low vs. high complexity), presentation order (high-complexity first vs. low-complexity first), and their interaction as fixed effects, with participant as a random intercept to correct for individual differences (repeated measures). To additionally check relationships between relevant dependent variables, Pearson correlations were computed among change scores (high–low) for the key eye metrics and DALI scores.

## 3. Results

### 3.1. Descriptive Results

Potential outliers were identified for all eye-tracking and subjective cognitive load variables using the interquartile range (*IQR*) method for the combined and condition-specific values [68]. Values falling more than 1.5 × *IQR* below the first quartile or above the third quartile were flagged as outliers. Only two participants showed outlier values in at least one measure. Identified outlier values (two for blink rate, one for DALI) were replaced with missing values (NA) to prevent undue influence on parameter estimation while maintaining the full dataset structure for subsequent analyses. See Table 1 for descriptive values.

### 3.2. Mixed-Effects Model

Separate linear mixed models were fitted for each eye-tracking parameter with condition (low vs. high cognitive load), presentation order, and their interaction as fixed effects, and participant as a random intercept.

Blink rate decreased significantly under high cognitive load (*b* = −1.49, SE = 0.57, *t* = −2.64, *p* = 0.010), indicating reduced blinking frequency when attentional demands increased. Neither presentation order nor the interaction reached significance (both *p* > 0.40). The intraclass correlation coefficient (ICC = 0.90) suggested substantial between-participant variability.

Fixation duration increased significantly in the high-load condition (*b* = 11.96, SE = 3.95, *t* = 3.03, *p* = 0.003), consistent with longer fixations under greater perceptual load. Neither order effects nor interactions were significant. Random-effects analysis showed an ICC of 0.87, indicating stable individual differences.

The model for fixation rate revealed a significant reduction under high load (*b* = −2.29, SE = 1.00, *t* = −2.29, *p* = 0.024), suggesting fewer fixations per time unit when perceptual load increased. The interaction between condition and order approached significance (*b* = 7.30, SE = 3.81, *t* = 1.92, *p* = 0.058), indicating a possible moderating influence of trial sequence. The ICC (0.89) again pointed to strong interindividual consistency.

**Figure 2:**
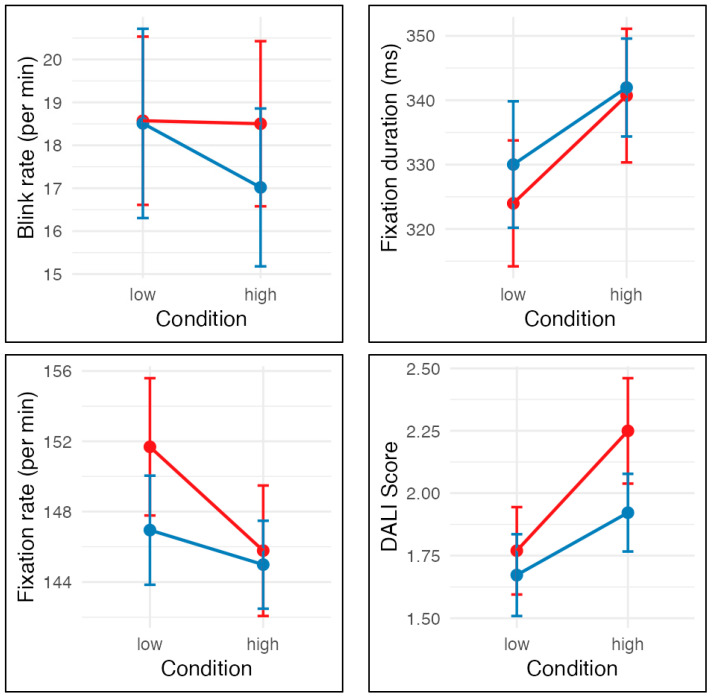
The figure displays the mean blink rate per minute (**top left**), fixation duration (**top right**), fixation rate (**bottom left**), and DALI scores (**bottom right**) across low and high conditions. Error bars represent ± one standard error of the mean. Red lines depict participants who drove the low-complexity condition first and then the high-complexity condition, whereas blue lines depict participants who experienced the high condition first and then the low condition.

Also, the DALI rating increased significantly in the high-load condition (*b* = 0.25, SE = 0.08, *t* = 3.15, *p* = 0.002). Neither order effects nor interactions were significant. Random-effects analysis showed an ICC of 0.89, indicating stable individual differences. See the model effects in Figure 2.

### 3.3. Correlation Analysis

While the mixed-effects models examined the differences between low- and high-complexity trips to compare conditions, the following correlation analysis focuses on the change values within participants to assess how individual changes are related across measurements. We performed the correlation analysis using the change scores of the key metrics (Table 2). A change score is the difference between the absolute values of the dependent variables in the high- and low-complexity condition and was calculated for each participant. The correlation analysis revealed fixation duration change correlated negatively with pupil change (*r* = −0.33, *p* < 0.05) and with DALI change (*r* = −0.29, *p* < 0.05), suggesting that an increase in metrics of cognitive load (DALI and pupil dilation) was associated with a decrease in fixation duration. Although fixation duration and DALI both increased at the condition level, correlations were computed on within-participant change scores (high–low), which may differ from effects observed in the mixed-effects models. Moreover, a decrease in fixation duration was also associated with an increase in blink rate (*r* = −0.30, *p* < 0.05) and an increase in fixation rate (*r* = −0.89, *p* < 0.001); other associations were nonsignificant.

## 4. Discussion

Understanding and monitoring drivers’ cognitive states remains a central challenge for traffic safety research, as lapses in attention, increased mental workload, and fatigue are strongly linked to impaired driving performance and elevated accident risk. Previous research has shown that cognitive workload is a multidimensional construct that cannot be fully captured by a single measurement approach, motivating the combined use of subjective, behavioral, and physiological indicators. While self-report instruments such as NASA-TLX are widely used to assess perceived workload, their sensitivity depends on the dominant task demands and they may fail to reflect rapid or involuntary changes in cognitive state. In response, eye-tracking has emerged as a particularly promising method for driving research, given the visual nature of the task and the close coupling between gaze behavior, attentional allocation, and mental workload. Importantly, previous studies suggest that the correspondence between subjective workload ratings and eye-movement measures depends on both the task characteristics and the workload instrument employed. This provides a relevant context for comparing driving-specific scales with domain-general measures in visually demanding tasks.

In contrast to Liu et al. [21], who reported that NASA-TLX and behavioral indicators primarily reflected cognitive rather than perceptual load, our results indicate that DALI likely captures perceptual demand more strongly. In our study, DALI scores increased with task complexity and correlated negatively with fixation duration, suggesting that participants’ subjective workload was driven mainly by perceptual strain—dense visual scenes and situational complexity—rather than cognitive effort. This pattern effectively mirrors Liu et al.’s findings in reverse: whereas their subjective workload aligned with cognitive processing, ours aligned with perceptual load, underscoring that the sensitivity of self-report scales depends on the dominant type of demand present in the task.

A likely explanation lies in the conceptual differences between DALI and NASA-TLX. While NASA-TLX was developed as a domain-general measure emphasizing mental, physical, and temporal demands along with effort, performance, and frustration, DALI was specifically designed for visually rich, dynamic tasks such as driving. It decomposes workload into more perceptual and sensorimotor dimensions, including vehicle operation and situational complexity, that capture the visual and attentional components of performance. Consequently, DALI may be inherently more sensitive to perceptual load, whereas NASA-TLX tends to emphasize cognitive effort and working-memory demands. The negative correlation between DALI and fixation duration observed in our study therefore reflects perceptual overload rather than cognitive strain, highlighting how the choice of subjective workload instrument shapes the interpretation of eye-tracking metrics.

Fixation durations were longer on average in the high-load condition. The increase in fixation duration observed under high-load conditions indicates deeper cognitive processing and sustained attentional engagement, suggesting that the task manipulation effectively increased cognitive demand. However, the negative correlation between fixation duration and DALI scores implies that participants’ subjective workload was more strongly influenced by perceptual factors—visual density and situational complexity—than by cognitive effort. Together, these findings suggest that while the experimental manipulation taxed both cognitive and perceptual resources, eye-movement data captured the cognitive component of workload, whereas DALI reflected the perceptual component.

In the study by Ktistakis et al. [5], two factors (time pressure and multitasking) were manipulated in a 2 × 2 design, resulting in four experimental activities: Activity 1 (no time pressure, single task), Activity 2 (time pressure, single task), Activity 3 (no time pressure, multitasking), and Activity 4 (time pressure, multitasking). Fixation frequency and pupil diameter emerged as the most sensitive eye-tracking indicators of workload, both showing significant effects of multitasking and time pressure. Specifically, fixation frequency decreased under multitasking but increased with time pressure, whereas pupil diameter increased in both conditions, indicating heightened arousal and effort. At the same time, fixation duration displayed an opposite trend: it increased during multitasking, reflecting greater cognitive engagement, but decreased under time pressure, consistent with more rapid visual scanning. When comparing activities of different difficulty levels (NASA-TLX order 1 < 2 < 3 < 4), fixation duration followed a different sequence (2 < 4 < 3 < 1), suggesting a non-linear relationship between subjective workload and gaze behavior. In particular, fixation duration increased from Activity 2 to 3, implying deeper processing as task demands rose, but then declined in Activity 4, where time pressure likely introduced perceptual overload and shortened fixations.

These patterns indicate that time pressure can be viewed as a form of visual complexity, eliciting faster scanning and reduced fixation duration, while multitasking primarily increases working-memory load, producing longer fixations and lower fixation frequency. Thus, fixation-based metrics differentiate between perceptual and cognitive components of workload, even when subjective NASA-TLX scores increase under both conditions. These distinctions align closely with our results: while fixation metrics differentiated cognitive and perceptual load in a manner consistent with the NASA-TLX patterns reported by Ktistakis et al. [5], DALI was more strongly driven by perceptual strain. This reinforces that DALI is uniquely tuned to visual density and situational complexity, making it more responsive to perceptual load than to cognitive effort.

Taken together, our findings demonstrate that in visually complex driving tasks, fixation-based eye-movement metrics and DALI ratings capture complementary but distinct components of mental workload, with DALI primarily reflecting perceptual demands rather than cognitive effort.

### Limitations

Several limitations should be considered when interpreting the present findings. First, the participant sample was relatively young and consisted predominantly of individuals with little driving experience, particularly with BEVs. Age and driving experience are known to influence visual attention strategies and perceived workload in complex driving situations, which may affect subjective ratings and eye-movement patterns. Therefore, the present results may not fully generalize to older or more experienced drivers. Moreover, the numbers of male and female participants are not balanced due to the unpredictability and high number of drop-outs. This limits the representativeness of the sample and increases the possibility that the observed effects do not apply equally to all genders.

The high number of excluded datasets constitutes a significant limitation of the study. During data collection, the eye-tracking system had to be replaced because the initial setup produced a substantial amount of invalid data due to recording failures and missing samples. Consequently, most exclusions were attributable to technical issues rather than participant-related factors. Data collected after the system change were retained for analysis, as the technical problems had been resolved and no further systematic causes for data loss were identified. Nevertheless, the change in hardware introduces a potential source of heterogeneity that should be considered when interpreting the results.

Because the study was conducted in a driving simulator, external validity is limited and potential confounding by simulator sickness must be considered. Participants who quit the experiment due to simulator sickness were excluded from analyses; however, discomfort could still have influenced subjective ratings and oculomotor behavior. Future studies might assess simulator sickness in all participants (e.g., SSQ) and test whether controlling for it alters the observed relationships. Also, more naturalistic study designs (e.g., on-road field studies) could address these simulator-specific confounders and improve external validity.

Conversely, future simulator studies could further increase experimental control to isolate specific physiological mechanisms more clearly. In the present study, pupil diameter was analysed exploratively because no pre-task baseline was available and luminance was not strictly controlled. In a 180° field-of-view simulator, effective retinal illumination varies with individual driving trajectories and gaze behaviour, potentially introducing noise into pupil-based workload measures. Implementing stricter luminance control or logging, together with baseline-corrected pupil measures, would allow a more precise assessment of pupil-related workload effects.

## 5. Conclusions

This study shows that in visually complex driving tasks, subjective workload ratings and eye-movement measures reflect different components of mental workload. Fixation-based metrics primarily captured cognitive processing demands, as indicated by longer fixation durations under higher task load. In contrast, DALI ratings were more strongly driven by perceptual strain, reflecting visual density and situational complexity rather than cognitive effort. These findings demonstrate that domain-specific workload instruments such as DALI are not interchangeable with domain-general measures like NASA-TLX, as they emphasize different underlying workload components. Combining eye tracking with driving-specific subjective scales therefore provides a more precise and interpretable assessment of mental workload in dynamic driving environments.

## Figures and Tables

**Table 1:** Descriptive statistics for eye-tracking and subjective cognitive load measures after outlier exclusion (*N* = 50).

Measure	Low Complexity		High Complexity	
	*M* (*SD*)	*N*	*M* (*SD*)	*N*
Blink Rate (per min)	18.5 (10.5)	49	17.7 (9.3)	49
Blink Duration (ms)	265.8 (27.8)	50	263.2 (28.1)	50
Fixation Rate (per min)	149.0 (17.3)	50	145.3 (15.0)	50
Fixation Duration (ms)	327.4 (49.0)	50	341.4 (43.7)	50
Pupil Diameter (mm)	3.7 (0.4)	50	4.0 (0.5)	50
DALI (0–5)	1.7 (0.8)	49	2.1 (0.9)	50

Values represent means (*M*) and standard deviations (*SD*) after exclusion of outliers using the 1.5 × *IQR* rule. *N* varies slightly due to missing values introduced by outlier imputation.

**Table 2:** Pearson correlations among eye-tracking metric change scores and DALI subjective cognitive load change scores.

Variable	1	2	3	4	5	6
1. Blink Rate Change	—					
2. Blink Duration Change	0.04	—				
3. Fixation Rate Change	0.09	0.09	—			
4. Fixation Duration Change	−0.30 *	−0.12	−0.89 ***	—		
5. Pupil Diameter Change	0.00	0.01	0.25	−0.33 *	—	
6. DALI Change	0.11	0.04	0.25	−0.29 *	−0.05	—

* *p* < 0.05, *** *p* < 0.001.

## Data Availability

The data presented in this study are available on request from the corresponding author due to the multimodal nature of the dataset and its fragmentation across multiple internal storage systems. These characteristics prevent the provision of a consolidated, self-contained public dataset. Data will be made available upon reasonable request, accompanied by support to ensure adequate reuse.
